# Alternative Splicing and Cancer

**DOI:** 10.1155/2012/363809

**Published:** 2012-05-20

**Authors:** Didier Auboeuf, Maria Carmo-Fonseca, Juan Valcarcel, Giuseppe Biamonti

**Affiliations:** ^1^Inserm U1052, CNRS UMR5286, Centre de Recherche en Cancérologie de Lyon, Université de Lyon, 69008 Lyon, France; ^2^Instituto de Medicina Molecular, Faculdade de Medicina, Universidade de Lisboa, Lisboa, Portugal; ^3^Institució Catalana de Recerca i Estudis Avançats (ICREA) and Centre de Regulacio Genomica, Barcelona, Spain; ^4^Istituto di Genetica Molecolare-Consiglio Nazionale delle Ricerche, Pavia, Italy

Alternative splicing of premessenger RNAs is a key step in the gene expression process, which allows the synthesis of different products from the same gene and contributes to increase the complexity of the proteome coded by a limited number of genes. Specialized high-throughput technologies (RNA-Seq, splicing-sensitive microarrays) aiming at analyzing alternative splicing in normal or pathological situations have allowed to make a promising step forward in basic and translational molecular oncology by identifying a variety of cancer-associated splicing variants. However, modification of alternative splicing is among the myriad of alterations present in cancer cells and whether splicing alteration is a cause or a consequence of cancer remains to be elucidated.

The main focus of this special issue is to highlight some of the mechanisms involved in splicing alteration in cancer and to present new evidence demonstrating the involvement of alternative splicing alterations in different steps and aspects of cancer initiation and progression.

To highlight the applications of large-scale approaches in the search for relevant cancer-associated splicing events, S. Germann and colleagues give an overview of the studies that have been carried out so far using such strategies. This has allowed to identify sets of functionally related genes whose expression is altered at the splicing level in cancer cells and to characterize some of the factors which control specific splicing programs that are deregulated in tumors.

The other reviews give a series of specific examples of cancer-associated splicing variants. S. Druillennec and colleagues address how alternative splicing modifies the physiological and pathological functions of a variety of protein kinases. Taking several examples of membrane-associated or cytosolic kinases, they explain more particularly how the oncogenic properties of this important class of factors between specific splicing isoforms.

More specifically, K. Holzmann and colleagues summarize the various splicing alterations that affect, in different tumor types, the transcripts encoding the fibroblast growth factor receptors (FGFR) 1–3, at the level of their IgIII loop. Splicing-induced variations in this domain, which occur naturally during embryonic development and are regulated in a tissue-specific manner, directly affect the interactions between the receptors and their ligands and have profound consequences on their activity. In cancer cells, alterations in FGFR2 splicing are involved in the epithelial-mesenchymal transition, an important step in the formation of metastases.

In their paper, Hilmi and colleagues focus on the alternative splicing of vascular endothelial growth factor (VEGF), which produces isoforms with opposite functions in the control of angiogenesis, a process involved in the progression and metastasis of several cancers. They discuss the emerging possibility of targeting angiogenesis more accurately by modulating the splicing of VEGF transcripts through the factors that control it.

J. Wei and colleagues review our current knowledge of the functional diversity of protein isoforms that compose the p53 family, encoded by the *TP53*, *TP63,* and *TP73* genes. Evidence shows that the aberrant expression of some of these isoforms in cancer cells contributes to tumor progression and may explain in some cases the resistance of tumors to therapeutic treatments.

Altogether those review articles illustrate how cancer cells may divert the natural properties of tissue-specific or developmentally regulated splicing variants to alter cellular functions and lead to a malignant phenotype or to a propagation of the tumor. This also underlines the need for a better understanding of the molecular mechanisms that control such splicing alterations.

*Didier Auboeuf* *Didier Auboeuf* *Maria Carmo-Fonseca* *Maria Carmo-Fonseca* *Juan Valcarcel* *Juan Valcarcel* *Giuseppe Biamonti* *Giuseppe Biamonti*

